# A type-5 metabotropic glutamate receptor-perineuronal net axis shapes the function of cortical GABAergic interneurons in chronic pain

**DOI:** 10.1186/s44158-025-00228-z

**Published:** 2025-02-21

**Authors:** Giada Mascio, Ferdinando Nicoletti, Giuseppe Battaglia, Serena Notartomaso

**Affiliations:** 1 IRCCS Neuromed, Pozzilli, Italy; 2 Department of Physiology and Pharmacology, Sapienza University of Rome, Rome, Italy

**Keywords:** Pain, Perineuronal nets, Metabotropic glutamate receptors 5

## Abstract

*Parvalbumin*-positive (PV^+^) interneurons (basket and chandelier cells) regulate the firing rate of pyramidal neurons in the cerebral cortex and play a key role in the generation of network oscillations in the cerebral cortex. A growing body of evidence suggest that cortical PV^+^ interneurons become overactive in chronic pain and contribute to nociceptive sensitization by inhibiting a top-down analgesic pathway. *Here, we provide further support to this hypothesis showing that intracortical infusion of the GABA*_*A*_* receptor antagonist, bicuculline, caused analgesia in a mouse model of chronic inflammatory pain, although it reduced pain thresholds in healthy mice*. We propose that mGlu5 metabotropic glutamate receptors and perineuronal nets (PNNs) shape the activity of PV^+^ interneurons in chronic pain, generating a form of maladaptive plasticity that enhances behavioural pain responses. mGlu5 receptors might be locally targeted by drugs activated by light delivered in cortical regions of the pain matrix, whereas the density of PNNs enwrapping PV^+^ interneurons might be reduced by local activation of PNN-degrading enzyme, such as type-9 matrix metalloproteinase. These strategies, which may require invasive treatments, might be beneficial in the management of severe pain which is refractory to conventional pharmacological and non-pharmacological interventions.

## Introduction

Nociceptive sensitization reflects the development of a maladaptive form of neuronal plasticity occurring in all stations of the pain neuraxis and *results into* increasing pain sensation and behavioural reactivity in response to sustained painful stimuli [1–5]. The study of the mechanisms underlying nociceptive sensitization is moving from single synapses to neuronal circuits connecting the regions of the pain matrix. Alterations of functional connectivity involving the salience network and the default mode network underlie suffering and embodiment of pain [6], which severely compromise the quality of life of patients with chronic inflammatory and neuropathic pain. Unravelling the mechanisms underlying abnormalities in network activity and functional connectivity may gain new insights into the pathophysiology of nociceptive sensitization and lay the groundwork for the design of novel therapeutic strategies in chronic pain. Besides their established role in pain perception and the emotional aspects of pain, the somatosensory cortex and the medial prefrontal cortex are involved in the regulation of pain thresholds by influencing the activity of brainstem nuclei that mediate the top-down control of pain [7]. Cortical network oscillations are generated and tuned by inhibitory GABAergic interneurons, which account for approximately 15% of the whole neuronal population in the cerebral cortex. There are different types of cortical interneurons, differentiated by their shape, location in the cortical layers, firing rate, and protein/peptide markers. *Parvalbumin*-positive (PV^+^) interneurons (basket and chandelier cells) and somatostatin-positive (SSt^+^) interneurons (Martinotti and non-Martinotti cells) originate from the medial ganglionic eminences and account for 70% of all interneurons, whereas the remaining interneurons originate from the caudal ganglionic eminences [8, 9]. Abnormalities of GABAergic interneurons have been described in models of chronic pain. For example, neuropathic pain is associated with an increased activity of PV^+^ interneurons and a reduced activity of SSt^+^ interneurons and vasoactive intestinal peptide (VIP)-expressing interneurons in the medial prefrontal cortex [10–12], whereas activation of SSt^+^ interneurons in the somatosensory cortex prevents the development of neuropathic pain [13]. Here, we focus on PV^+^ interneurons, which are fast-spiking, make synapses with the cell body and axon initial segment of pyramidal neurons, and play a key role in the regulation of network oscillations [14]. We discuss how type-5 metabotropic glutamate (mGlu5) receptors and perineuronal nets (PNNs) shape the activity of PV^+^ interneurons and might contribute to nociceptive sensitization in chronic inflammatory and neuropathic pain.

### mGlu5 receptors and chronic pain

mGlu5 receptors are coupled to G_q/11_, and their activation stimulates polyphosphoinositide (PI) hydrolysis with ensuing formation of inositol-1,4–5-trisphosphate and diacylglycerol [15]. mGlu5 receptors are expressed in most regions of the pain neuraxis, from peripheral nociceptors to the regions of the pain matrix encoding the sensory, affective, and emotional aspects of pain, and contribute to the development of nociceptive sensitization through a plethora of CNS region-specific mechanisms. For example, activation of mGlu5 receptors in nociceptors enhances the activity of peripheral pain transducers (i.e. TrpV1 receptors) through a chain of reactions that involve intracellular calcium release, activation of cyclooxygenase, formation of prostaglandins, autocrine/paracrine activation of prostaglandin receptors, stimulation of cAMP formation, activation of protein kinase A, and TrpV1 phosphorylation [16]. mGlu5 receptors are highly expressed by second-order sensory neurons in the dorsal horns of the spinal cord, where their activation enhances neuronal responsiveness to synaptic inputs [17–19]. In their pioneer study, Robert Gereau 4th and his associates have found that activation of mGlu5 receptors in dorsal horn sensory neurons induces nociceptive sensitization through a mechanism involving activation of the extracellular-regulated kinase pathway and inhibition of K_v_4.2 potassium channels [20]. Interestingly, membrane recycling of mGlu5 receptors in dorsal horn neurons mediated by interaction with vacuolar protein sorting-associated protein 26 (VPS26) and sorting nexin 27 (SNX27) has been implicated in the development of neuropathic pain in rats [21]. Chronic pain is also associated with changes in the expression and/or function of mGlu5 receptors in supraspinal centres of the pain pathway. Positive emission tomography studies with the mGlu5 tracer, [^11^C]ABP688, in rats have shown that spinal nerve injury caused an upregulation of mGlu5 receptors in the prelimbic region of the medial prefrontal cortex, and pharmacological blockade of mGlu5 receptors in the prelimbic cortex attenuated neuropathic pain and associated negative mood symptoms [22]. Volker Neugebauer and his associates have shown that arthritic pain disrupts the ability of mGlu5 receptors to engage endocannabinoid signaling and restrain synaptic inhibition in layer-5 pyramidal neurons of the infralimbic cortex. This maladaptive form of synaptic plasticity prevents mGlu5 receptors from activating an output pathway that attenuates pain behaviours and cognitive deficits [23]. Changes in mGlu5 receptor signaling have been found in regions of the pain matrix in response to unilateral chronic constriction injury (CCI) of the sciatic nerve, with mGlu5-mediated PI hydrolysis being up-regulated in the contralateral prelimbic, infralimbic and cingulate cortices, and basolteral amygdala [24]. mGlu5 receptors functionally interact with CB1 cannabinoid receptors in the periaqueductal grey and rostral ventromedial medulla, the two main brainstem centres involved in the top-down control of pain [25, 26]. *In addition, Binn and Salt explored the possible role of mGlu5 in thalamic nociceptive responses* [27, 28], *also showing EEG effects of the mGlu5 blocker*, *2-methyl-6-(phenylethynyl)pyridine (MPEP), in addition to activity on thalamic responses*.

The role of mGlu5 receptors in the regulation of pain thresholds has been extensively studied using selective mGlu5-negative allosteric modulators (NAMs), which inhibit receptor activity regardless of the ambient concentrations of glutamate and are systemically active owing to their high hydrofobicity. mGlu5 receptor NAMs have consistently shown robust analgesic activity in models of inflammatory or neuropathic pain [24, 29–44]. However, preclinical to clinical translation of mGlu5 receptor NAMs has been disappointing [45], suggesting that the effect of these drugs in the various stations of the pain neuraxis is not homogenous.

Light-sensitive mGlu5 receptor NAMs have provided a powerful tool for the identification of brain regions that are sufficient and/or necessary for the induction of analgesia induced by systemic mGlu5 receptor blockade. Light-induced activation of a caged derivative of the mGlu5 receptor NAM, raseglurant (compound JF-NP-26), in the thalamus caused analgesia in the chronic constriction injury (CCI) model of neuropathic pain and in a mouse model of cancer breakthrough pain [24, 43, 44]. Transcutaneous illumination of JN-NP-26 also caused analgesia in both phases of the formalin test in mice [43]. As opposed to JF-NP-26, which is activated by light [43], the mGlu5 receptor NAM alloswitch-1 is active on its own and is *inactivated* by light at 405 nm [46]. The use of these two compounds allowed to establish that mGlu5 receptor blockade in the thalamus and medial prefrontal cortex is both necessary and sufficient for the induction of analgesia in response to systemic mGlu5 receptor NAMs, whereas receptor blockade in the basolateral amygdala enhanced pain responses in neuropathic mice [24].

What is particularly relevant for this viewpoint is that mGlu5 receptors are highly expressed by PV^+^ GABAergic interneurons [47] and are involved in mechanisms of activity-dependent synaptic plasticity in cortical fast-spiking of PV^+^ interneurons [48].

**Fig. 1:**
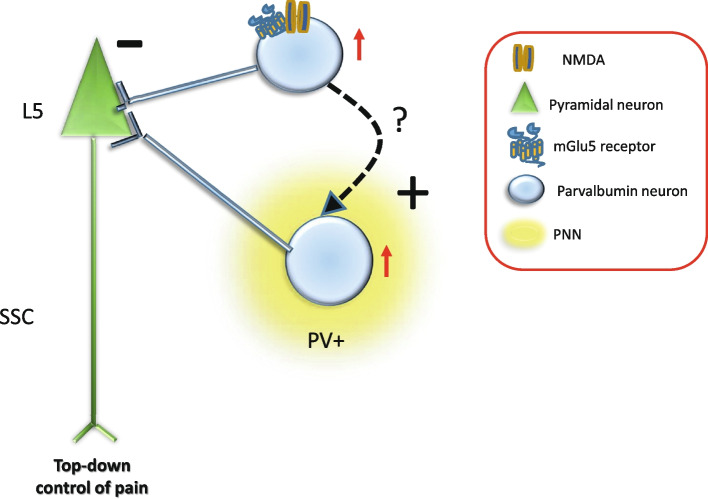
Activation of mGlu5 receptors and formation of PNNs may caused a novel activity of PV^+^ interneurons, which in turn inhibit a top-down analgesic pathway originated from layer 5 of SSCtx. The interconnection between activation of mGlu5 receptors and formations of PNNs remains to be elucidated

Cell-specific deletion of mGlu5 receptors in PV^+^ interneurons reduced inhibitory currents and impaired cortical network oscillations activity [49]. mGlu5 receptors are also involved in the development PV^+^ interneurons, as shown by a reduced expression of PV in the cerebral cortex and hippocampus of mGlu5 receptor knockout mice [50]. Interestingly, mGlu5 receptors are co-expressed and interact with NMDA receptors [51, 52], which support the activity of PV^+^ interneurons [53]. We suggest that cortical mGlu5 receptors contribute to the development of nociceptive sensitization by supporting the activity of PV^+^ interneurons, which, in turn, negatively regulate a top-down analgesic pathway originating from layer-5 pyramidal neurons (Fig. 1).

### PNNs and maladaptive plasticity of cortical interneurons in chronic pain

PNNs, which are condensed structures of the extracellular matrix formed by chondroitin sulphate proteoglycans linked to hyaluronic acid and tenascin-R, surround PV^+^ interneurons and other types of GABAergic interneurons, in the cerebral cortex and other CNS regions. Their formation coincides with the closure of temporal windows of cortical plasticity during postnatal development [54–56], and this isolates PV^+^ interneurons from the action of trophic factors, which might have detrimental effects on their function and plasticity. However, PNNs are dynamic structures, which may disassemble and assemble in response to stress or during learning [57, 58]. *Dysfunction in PV/PNNs interneurons in thalamus and cortex has been implicated in a number of other conditions in psychiatry and neurology* [59]*.*

**Fig. 2:**
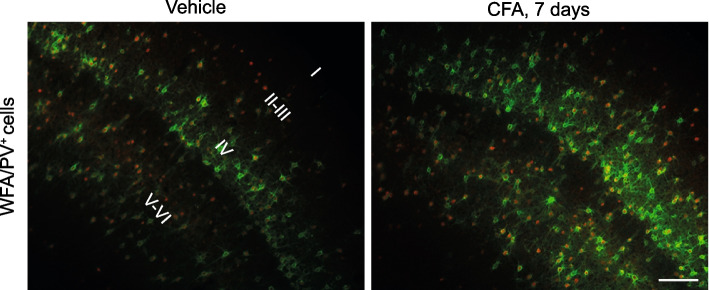
Increased density of WFA^+^ PNNs in the SSCtx of mice developing chronic inflammatory pain. Representative images showing the increased density of WFA^+^ PNNs (green) and PV^+^ neurons (red) in the contralateral SSCtx associated with chronic inflammatory pain (Mascio et al., [60]). Scale bar 100 µm

We found recently that chronic inflammatory pain in mice induced by unilateral injection of complete Freund adjuvant (CFA) in the hind paw was associated with an increased density of PNNs labelled with the lectin, *Wisteria floribunda* (WFA), in the contralateral somatosensory cortex, medial prefrontal cortex (e.g. anterior cingulate, prelimbic, and infralimbic cortices), and reticular thalamic nuclei [60] (Fig. 2).

**Fig. 3:**
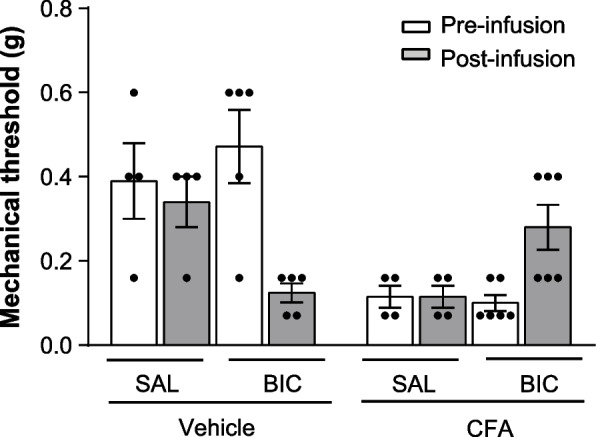
Effect of intracortical infusion of bicuculline on pain thresholds in mice injected with CFA or vehicle. Mice were anaesthesized with isoflurane and stereotaxically implanted with a 27-gauge injection cannula in the SCCtx (*AP*: 0, *L*: 3, *DV* 2.2 mm), fixed with acrylic cement. Three days later, mice were injected with CFA or vehicle in the hindlimb contralateral to the injection cannula. Bicuculline (Tocris Cookson Ltd., Bristol, UK) was dissolved into saline and infused in the SSCtx contralateral to CFA or vehicle with a 5-µl Hamilton syringe connected by a catheter to the injection cannula. Mice were infused with 0.5 µl of bicuculline solution (20 ng/µl) or saline in 90 s. Mechanical thresholds were measured 1 h prior to and 5 min after intracortical infusions. Values are means ± SEM of 4–6 mice per group. *Significantly different vs. the respective values obtained 1 h prior to bicuculline infusion. The experimental protocol was approved by the Italian Ministry of Health, 1135/2020-PR

Increases in PNN density were also found in different regions of the pain matrix after induction of neuropathic pain (Mascio et al., under revision). In mice with chronic inflammatory pain, local enzymatic degradation of PNNs in the somatosensory cortex caused analgesia and corrected the enhanced inhibitory transmission at the synapses between interneurons and layer-5 pyramidal neurons associated with chronic pain [60]. This discloses a novel form of maladaptive plasticity in which an increased formation of PNNs stabilizes excitatory inputs of PV^+^ interneurons [61], which become overactive and restrain the activity of a descending analgesic pathway. This hypothesis was supported by experiments in which the GABA_A_ receptor antagonist, bicuculline, was injected in the mouse somatosensory cortex. In control mice, GABA_A_ receptor blockade in the somatosensory cortex reduced pain thresholds, as expected. Opposite effects were seen in mice with chronic inflammatory pain, in which GABA_A_ receptor blockade in the somatosensory cortex caused analgesia (Fig. 3, unpublished data, methods and statistics are reported in the figure legend).

Activation of mGlu5 receptors and enhanced formation/reduced degradation of PNNs may converge to increase the activity of PV^+^ interneurons, which, in turn, inhibit a descending analgesic pathway, as outlined above. What remains to be established is whether these two phenomena, i.e. activation of mGlu5 receptors and the build-up of PNNs enwrapping GABAergic interneurons associated with chronic pain, are interconnected. This question has not been addressed in models of chronic pain. However, we found that mGlu5 receptors are key regulators of PNN formation during the first 16 days of postnatal development, when genetic deletion or pharmacological blockade of mGlu5 receptors substantially enhance the density of PNNs in the somatosensory cortex. This effect was associated with an increased expression of genes encoding structural components of PNNs and a reduced activity of the PNN-degrading enzyme, type-9 matrix metalloproteinase (MMP-9) [62]. In addition, mGlu5 receptors shaped the PNN response to sensory stimulation or deprivation in the first 2 weeks of postnatal life [62]. Interestingly, mGlu5 receptors are highly expressed and functional in the first 2 weeks of postnatal life, when mGlu5 receptor-mediated PI response is remarkable [63–67].

Although the density of PNNs in the adult somatosensory cortex did not differ between wild-type and mGlu5 knockout mice [62], we cannot exclude that the control of mGlu5 receptors on PNN formation is restored within the context of nociceptive sensitization and perhaps in an opposite direction with respect to early postnatal development. It will be interesting to examine whether the build-up of PNNs associated with chronic pain in difference regions of the pain matrix is affected by treatment with drugs that either activate or inhibit mGlu5 receptors.

## Concluding remarks

In spite of the multiple classes of analgesic agents, the treatment of chronic pain is still suboptimal, and some types of pain, e.g. neuropathic pain, are highly resistant to medication [68, 69]. The social and economic burden is enormous considering that 15 − 20% of visits at physicians are caused by chronic pain [68]. Novel treatments are urgently needed, and the use of drugs targeting specific cell types of the pain matrix regulating pain perception and motor and affective responses to pain is a path to follow. The use of transcranial magnetic stimulation (TMS) is a remarkable example of a brain region-specific approach in the treatment of refractory pain [70–72]. Interestingly, modulation of GABAergic transmission has been proposed as one of the mechanisms by which repetitive TMS is beneficial in the treatment of pain [73].

Here, we propose a novel mechanism of nociceptive sensitization based on structural and functional changes in PV^+^ interneurons in different regions of the pain matrix. mGlu5 receptors, which become highly functional in response to pain (see above), might synergize with NMDA receptors in activating PV^+^ interneurons, thus disrupting a defensive mechanism against pathological pain. In addition, activation of mGlu5 might drive a chain of intracellular reactions that either enhance the formation or reduce degradation of PNNs, which would stabilize the activity of PV^+^ interneurons. While the causal relationship between PNNs and behavioural responses to chronic inflammatory or neuropathic pain has been established ([60], Mascio et al., under revision], the link between mGlu5 receptors and PNNs is still speculative and remains to be demonstrated. What makes this mechanism attractive is that it can be targeted by therapeutic intervention according to the principle of precision medicine. The use of mGlu5 receptor NAMs in the treatment of chronic pain could be optimized if these drugs are specifically directed towards regions of the pain matrix where receptor blockade is sufficient and necessary to cause analgesia. This could be achieved by light-sensitive drugs, which can be activated in a specific brain region with high spatio-temporal resolution [43, 44]. This intervention requires the implantation of LEDs in the brain parenchyma and, therefore, might be restricted to severe types of pain that are refractory to medication.

Targeting PNNs in the treatment of pain is a more difficult task for two reasons: (i) it requires the local injection of chondroitinase or other PNN-degrading enzyme in the somatosensory cortex, which is not practical from a therapeutic standpoint, and (ii) this intervention might disrupt the physiological function of the extracellular matrix, and, therefore, disrupt an important mechanism in the regulation of synaptic function. It may be interesting, however, to examine whether, and to what extent, different classes of analgesic drugs affect the activity of enzymes that regulate the turnover rate of PNNs, such as MMP-9. These drugs might be locally delivered (or activated by light) to limit the build-up of PNNs associated with chronic pain.

## Data Availability

No datasets were generated or analysed during the current study.

## Abbreviations

PV^+^: Parvalbumin-positive interneurons
PNNs: Perineuronal nets
SSt^+^: Somatostatin-positive interneurons
VIP: Vasoactive intestinal peptide-expressing interneurons
mGlu5: Type-5 metabotropic glutamate receptors
PI: Polyphosphoinositide hydrolysis
VPS26: Vacuolar protein sorting-associated protein 26
SNX27: Sorting nexin 27
CB1: Type-1 cannabinoid receptors
NAMs: Negative allosteric modulators
CCI: Chronic constriction injury
CFA: Complete Freund adjuvant
WFA: *Wisteria floribunda*
MMP-9: Type-9 matrix metalloproteinase
TMS: Transcranial magnetic stimulation
